# Physical home learning environments for digitally-supported learning in academic continuing education during COVID-19 pandemic

**DOI:** 10.1007/s10984-022-09406-0

**Published:** 2022-02-24

**Authors:** Filiz Keser Aschenberger, Gregor Radinger, Sonja Brachtl, Christina Ipser, Stefan Oppl

**Affiliations:** 1 Donau-Universität Krems, Krems, Austria; 2 Department for Continuing Education Research and Educational Technologies, Danube University Krems, Dr.-Karl-Dorrek-Straße 30, 3500 Krems, Austria; 3 Department for Building and Environment, Danube University Krems, Dr.-Karl-Dorrek-Straße 30, 3500 Krems, Austria

**Keywords:** COVID-19 pandemic, Distance learning, Home learning environment, Non-traditional students, Physical learning space, Well-being

## Abstract

Since the outbreak of the COVID-19 pandemic in 2020, digital technologies for distance learning have been used in educational institutions worldwide, raising issues about social implications, technological development, and teaching and learning strategies. While disparities regarding access to technical equipment and the internet (‘the digital divide’) have been the subject of previous research, the physical learning environment of learners participating in online learning activities has hardly been investigated. In this study, the physical-spatial conditions of learning environments, including technical equipment for distance learning activities and their influence on adult learners in academic continuing education during initial COVID-19 restrictions, were examined. Data were collected with an online survey sent to all students enrolled in an Austrian continuing education university, together with a small number of semi-structured interviews. A total of 257 students participated in the survey during the 2020 summer semester. Our findings provide insights in two infrequently-studied areas in learning environment research: the physical learning environment for online learning and the learning environment in academic continuing education. The study illustrates that students in academic continuing education have spacious living conditions and almost all the equipment necessary for digitally-supported learning. According to gender and household structure, significant differences were found regarding technical equipment, ergonomic furniture and availability of a dedicated learning place. In their learning sessions during the restrictions, students reported low stress levels and positive well-being. The more that they perceived that their physical learning environment was meeting their needs, the higher were their motivation and well-being and the lower was their stress. Their learning experience was further improved by the extent to which they had a separate and fixed learning place that did not need to be coordinated or shared with others. The study contributes to the literature on creating conducive learning environments for digitally-supported online learning for adult learners.

## Introduction

The COVID-19 pandemic has caused rapid and radical changes in the way we live, work and learn throughout 2020 and beyond. On March 11, 2020, the World Health Organization (WHO) officially declared the SARS-CoV-2 virus to be a global pandemic. Since then, all countries in the world have been taking measures to mitigate the risks and deal with the challenges of this pandemic. The closure of schools, universities and all other educational institutions was among the first measures taken. In April 2020, most of the educational institutions worldwide were shut down, leaving 1.6 billion students in 188 countries without access to education (Gouëdard et al., 2020). In 109 countries, 59% of higher-education institutions halted all campus activities and were completely shut down (Marinoni et al., 2020). Almost all countries switched to distance learning for all levels of education, using a variety of tools and platforms such as television, synchronous courses through online meeting platforms, and asynchronous lectures via learning platforms, etc. In higher education, the majority of the institutions replaced classroom learning with distance teaching and learning. However, not every country possessed the means and resources to adjust to distance learning. While 85% of the institutions in Europe replaced in-person teaching with distance learning, only 29% of African institutions were able to adapt to the requirements of distance learning and teaching (Marinoni et al., 2020). The discrepancies and inequalities observed at global and institutional levels are strongly reflected in household and individual levels.

One of the greatest concerns regarding the impact of the COVID-19 pandemic on education has been the exclusion of learners from vulnerable groups that have no or limited access to the technical equipment and internet connectivity required for participating in distance learning. This “gap between individuals, households, businesses and geographic areas at different socio-economic levels with regard both to their opportunities to access information and communication technologies (ICTs) and to their use of the internet for a wide variety of activities” (OECD, 2001) has been labelled as the ‘digital divide’. Although the concept has gained broader meaning to encompass social inclusion and participation with the recent work of researchers such as Van Dick (2015), for this study, it can be operationalised in terms of accessibility and affordability of ICT equipment (computers, tablets, smartphones) and the internet. At a time when the majority of learning activities are conducted via distance learning, having access to basic ICT equipment and the internet is crucial to enable students to participate in learning activities. However, an at-home physical learning environment conducive to academic work is as important as internet access and ICT equipment for the well-being and learning activities of students of all ages (Di Pietro et al., 2020). While the digital divide (DiMaggio et al., 2004) and the related inequities regarding internet access and ICT devices have been some of the main concerns, especially for learners with disadvantaged backgrounds, the physical learning environment in which people are situated while participating in online learning activities has hardly been raised as a concern.

Learning environment research, which dates back to the work of Herbert Walberg and Rudolf Moos (Fraser, 2018), has been increasing significantly, particularly in regard to the psychosocial learning environment in formal learning spaces such as classrooms and laboratories. Recent conceptualisations of learning environments provide a more holistic perspective including the physical and technological aspects and informal learning spaces outside classrooms and schools (Manninen et al., 2007; Radcliffe et al., 2009; Valtonen et al., 2021).

In this article, we focus on the physical aspects of the learning environment in an informal learning setting. The impact of physical learning space on different aspects of learning for both compulsory and post-compulsory education (e.g. satisfaction, achievement and engagement) has been well-established and recognised in educational sciences as well as in design and architecture (Barrett et al., 2013; Choi et al., 2014; Han et al., 2019; Higgins et al., 2005; Sivunen et al., 2014; C. Wang et al., 2021; L. Xiong et al., 2018). Characteristics of the physical space are relevant not only for achieving the intended learning outcomes, but also for health and physical and mental well-being (Clark et al., 2007; Codinhoto et al., 2009; Cooper et al., 2009; Rashid & Zimring, 2008). There have also been a few significant studies on measuring the psychosocial learning environment in post-secondary distance education (Joiner et al., 2020; Walker & Fraser, 2005) and a recent project, ‘Onlife Learning Spaces’, by Ninnemann (2020), who examined the hybrid learning environments of a similar group of adult students.

Yet, physical learning environments for digitally-supported distance learning activities have not attracted significant attention to date. Furthermore, adult learners and their learning conditions have been overlooked in academic discussions. These learners in post-compulsory education are considered a special group with distinct needs and expectations because of their demographic backgrounds and are mostly identified as ‘non-traditional students’ or ‘mature students’, which can be defined as “students who are 25 years or older, attend part-time, are financially independent, have other major responsibilities and roles that compete with their studies (e.g. parenting, caregiving, employment and community involvement) and/or lack the standard admission requirements of a program” (Panacci, 2015, p. 2). In our case, they are studying at a continuing education university that offers Master and academic certificate degree programmes. Adult learners in post-compulsory education are usually confronted with three competing domains (work, family and studies) and the need to manage boundaries between them (Ahrentzen, 1990). While digitalisation has blurred the traditional boundaries between work and family life (Oppl et al., 2019) in recent decades, the COVID-19 pandemic has made it almost impossible to manage these boundaries because of lockdowns, school closures, and home-office regulations (Kossek et al., 2021). Therefore, it is crucial to examine the physical learning spaces and experiences of adult students during the pandemic to understand the role that learning environment plays for well-being and learning outcomes.

Baticulon and colleagues’ (2021) study of barriers to online learning during the COVID-19 pandemic in the Philippines indicated that limited workspace conducive to studying and mental health difficulties were among the major barriers for students’ participation in online learning. Another study by Kapasia et al. (2020) yielded similar results: 44% of the students did not have a separate room for studying; 42% reported feelings of stress, depression and anxiety; and 12.6% did not have a favourable environment for studying at home (pp. 3–4). In a similar study (Lister et al., 2021), physical learning space was identified as both a barrier and an enabler for a sense of well-being for students engaged in online learning. Moreover, a systematic review conducted by Wang et al. (2020) showed that one in three adults in the general population had COVID-19-related psychological distress, and that women, younger people, those of lower socioeconomic status (SES) and those living in rural areas were at higher risk for stress and anxiety. Yet, little is known about the home learning experiences of this specific group because of the lack of research focussing on adult learners in post-compulsory education, specifically in academic continuing education, and investigations of their learning environments and well-being during the COVID-19 pandemic.

## Current study

Based on this background, this study examined the spatial environments and technical equipment for distance learning processes and their influence on adult learners and learning activities in academic continuing education in order to shed light on learners’ experiences and on the inequalities and challenges that they have faced during the COVID-19 pandemic. To pursue this goal, the following research questions were delineated:

### Research Question 1

 Under which physical-spatial conditions (including technical equipment) does digitally-supported student learning in the academic continuing education sector take place during the initial COVID-19 restrictions?

### Research Question 2

How do students in the field of academic continuing education perceive their home learning environment for digitally-supported learning during the initial COVID-19 restrictions?

### Research Question 3

How do the physical-spatial conditions of students in academic continuing education differ according to gender, age, and household structure (with/without children)?

### Research Question 4

What influence do different physical-spatial conditions have on well-being and learning experience?

## Research design and methods

This study involved an online survey (Creswell & Creswell, 2018), which was followed by semi-structured interviews with a small number of students who volunteered to participate to explore the findings of the survey in greater depth and support the interpretation of the results.

### Research context

The study was conducted at Danube University Krems, Austria, which specialises in academic continuing education. It focusses solely on postgraduate education and, consequently, has a different student body compared with traditional higher-education institutions. Currently, about 8000 students are enrolled in the university’s study programmes. The average age of students is about 40 years, but the range of age groups is highly diverse: 19.4% of students are over 50 years and 2% are over 60 years of age. The majority of the students at Danube University Krems are employed while studying, with several years of work experience and, in most cases, management and leadership experience. Students have diverse educational backgrounds that can be divided into three broad groups: students with a higher education degree achieved in prior studies; students with a formal higher-education entrance qualification but no prior studies; and students without a formal higher-education entrance qualifications but equivalent qualifications achieved via non-formal or informal learning (Humer et al., 2019).

### Data collection

#### Online survey

The questionnaire for the online survey was developed through collaboration between education researchers, psychologists and architects to analyse learning experiences as well as the private, physical learning environments of students in the academic continuing education sector during the COVID-19 pandemic. We adopt the terms ‘physical learning environment’ and ‘learning place’ to refer to the physical environment that was predominantly used for online learning. The survey was created in German, with the term *überwiegend genutzter Lern- bzw. Arbeitsplatz* used to refer to the physical learning environment.

A pilot test was conducted to increase validity and reliability. The pilot-testing process was conducted with 10 students from Danube University Krems, with feedback regarding functionality, comprehensibility and time expenditure being collected and the survey being adapted accordingly. The web link to the survey, which was sent at the end of June 2020 to all students who were enrolled in Danube University courses in the summer semester of 2020, reached a total of 7737 people. The participation period ended on July 31st.

#### Structure of the questionnaire

The questionnaire comprised four blocks: (1) socio-demographic information as well as individual physical-spatial conditions of the home learning environment; (2) perceived fulfilment of the personal requirements for the physical learning environment; (3) psychological characteristics such as well-being, stress and motivation, measured with standardised questionnaires; and 4) learning experiences during the initial COVID-19-related restrictions. We delineated the factors to be included by using the literature.

1. Socio-demographic information and spatial characteristics of the home learning environment included: gender, age, study area, household type, household members, living environment (urban or rural), housing type, dwelling size and access to outdoor space, as well as characteristics of the physical learning environment, including previous existence of the learning place, the purpose of the room used for learning (one’s own room for studying or mixed use of the space), location for learning activities (always at the designated learning place or a changing location), availability of the learning place (whether its use had to be coordinated with use by others), furniture and IT equipment (Han et al., 2018; Hill & Epps, 2010; Hutchinson, 2003; Ramprasad & Subbaiyan, 2017).
2. Students’ perceptions of the fulfilled personal requirements of their predominantly-used learning place were measured by 11 attributes related to spatial characteristics and indoor environmental conditions that were found in previous studies to influence building occupants’ health and well-being as well as students’ learning performances and satisfaction: an adequate supply of daylight and a pleasant view (Tanner, 2009); comfortable temperature conditions, good ventilation, protection against noise pollution, and attractive interior design (Lee et al., 2012; Mujan et al., 2019; L. Xiong et al., 2018); a distraction-free environment, adequate size, ergonomic work-compatible furniture adaptable to individual spatial requirements (Hutchinson, 2003; Ramprasad & Subbaiyan, 2017); and the appropriate technical equipment (Han et al., 2018; Hill & Epps, 2010). The level of agreement with whether the personal requirements were met was measured on a 4-point scale of (1) disagree, (2) rather disagree, (3) rather agree and (4) agree.
3. Standardised questionnaires were used to measure participants’ well-being, stress, and motivation. **Well-being** was assessed by the WHO’s (Five) Well-being Index (WHO-5) (WHO Collaborating Centre in Mental Health, 1998). This questionnaire measures current mental well-being (with a time frame of the previous two weeks) and uses only positively-phrased questions to avoid symptom-related language. It contains five items rated on a 6-point scale and raw scores range from 0 to 25. A score below 13 represents poor well-being and is an indication for testing for depression under ICD-10. **Stress** was assessed using the Perceived Stress Questionnaire (PSQ, short version) (Fliege et al., 2009; Levenstein et al., 1993) which consists of 20 items and contains the four subscales of ‘worry’, ‘tension’, ‘joy’ and ‘demands’. An overall score (0–100) can be calculated from these subscales, with a higher score indicating a greater level of self-reported stress. **Motivation** was assessed using the four marker items of the LEIMO achievement motivation test rated on a 5-point scale (Guttschick, 2015). Based on reliability analysis, an overall score was calculated with the exclusion of Item 3 (which showed low item selectivity); a higher score indicates greater motivation. Detailed information about the reliability analysis of the questionnaires can be found in Table 1.
4. Learning experiences during the initial COVID-19-related restrictions were assessed by three questions (five response options): (1) Influence of the learning place (“The physical environment at my predominantly used learning place in the last four months influenced my ____ (motivation to learn, concentration, learning performance)” (6-point scale with two poles, negative–positive); (2) Well-being at the learning place (“I felt ____ (unwell / well) at my predominantly used learning place”) (6-point scale); and (3) Perceived suitability of the learning place (“I found my predominantly used learning place to be ____ (unsuitable/suitable)” (6-point scale).

**Table 1:** Reliability analysis of questionnaires (WHO-5, PSQ, LEIMO Marker Items)

Scale	Items *k*	*n*	Cronbach α	Selectivity (*r*_*it*_)	*M (SD*)
WHO-5 Well-Being (0–5)	5	253	0.89	0.63–0.78	15.02 (5.45)
PSQ overall (1–4; PR 0–100)	20	244	0.95	0.46–0.82	37.5 (21.5)
Worry	5	252	0.84	0.54–0.72	28.4 (22.5)
Tension	5	253	0.89	0.64–0.79	37.7 (25.7)
Joy	5	245	0.86	0.59–0.74	63.9 (24.1)
Demands	5	251	0.85	0.55–.0.72	46.9 (25.6)
LEIMO Motivation (1–5)	3	249	0.58	0.31–0.47	3.99 (0.75)

Rating scales for the instruments: WHO 5: (0) at no time, (1) some of the time, (2) less than half the time, (3) more than half the time, (4) most of the time, (5) all of the time; PSQ: (1) almost never, (2) sometimes, (3) often, (4) usually; LEIMO: (1) strongly disagree, (2) rather disagree, (3) partly agree (4) rather agree, (5) strongly agree

### Online interviews

After the online survey, semi-structured online interviews (Atteslander et al., 2010; Salmons, 2015) referring to the main topics of the online survey were conducted with seven students. These interviews were intended to describe individual living and learning situations in order to supplement the quantitative survey results with qualitative statements and to support the interpretation of results of the online survey. Invitations to participate in the interviews were sent by course directors in all three faculties of the university, with students who wished to take part contacting the researchers. The individual interviews conducted in the context of online video meetings were recorded and subsequently transcribed.

### Data analysis

#### Statistical analysis

Descriptive statistics were used to understand under which physical-spatial conditions students’ learning took place during the initial COVID-19 restrictions. Exploratory factor analysis (EFA) was used to identify possible dimensions that could explain the interrelationships between the 11 attributes of students’ perceptions of personal requirements for the physical home learning environment. Differences between the physical-spatial conditions according to gender, age and household structure were depicted with crosstabs and $\chi^{2}$ tests. The influence of different spatial characteristics on the perceived impact of the learning environment was analysed with independent *t*-tests. Furthermore, the impact of personal factors and physical-spatial conditions on students’ motivation, stress and well-being was measured using multiple regression analysis.

#### Interview analysis

The seven qualitative online interviews provided information about the physical conditions for learning, including the room and IT equipment, as well as subjective impressions regarding personal well-being and experiences during online learning activities. Thematic analysis (Howitt, 2016, p. 163) was used to explore the possible connections between the learning experiences and the physical learning environments of students of different ages and genders.

## Results

### Sample characteristics

#### Online survey participants

A total of 257 students completed the online questionnaire. Table 2 presents the sample characteristics. The majority of our participants were female (56.8%) and the average age was 40.5 years. At the time of the survey, about half of the participants had no children living in the household and almost two-thirds were living in multi-person households. Moreover, they lived in relatively-large flats or houses with outdoor spaces such as a garden or terrace. An interesting characteristic was that 54.5% of participants had no previous experience with online learning.

**Table 2:** Sample demographics and household characteristics

Demographic and household characteristics	*f*	%
*Gender *^*a*^		
Male	107	41.6
Female	146	56.8
Diverse	1	0.4
No indication made	3	1.2
*Age *^*a*^		
< 24 years	5	1.9
25–34 years	66	25.7
35–44 years	94	36.6
45–54 years	73	28.4
≥ 55 years	17	6.6
No indication made	2	0.8
*Experience with online learning *^*a*^		
No previous experience	140	54.5
Previous experience	117	45.5
*Household form* ^a^		
Multi-person household	199	77.4
One-person household	50	19.5
Shared apartment	4	1.6
No indication made	4	1.6
*Household structure *^*b*^		
No children in the household	136	52.9
Child/ren of compulsory school age	54	21
Child/ren of preschool age	38	14.8
Child/ren no longer of compulsory school age	23	8.9
Household with more than two generations	16	6.2
Household with pets	77	30
No indication made	4	1.6
*Living environment *^*a*^		
Urban	85	33.1
Suburban	63	24.5
Village	72	28
Rural	35	13.6
No indication made	2	0.8
*Residential building type *^*a*^		
Detached single-family house`	108	42
Semi-detached or terraced house	19	7.4
Multi-party house	114	44.4
No indication made	16	6.2
*Flat size *^*a*^		
< 40 m^2^	15	5.8
40 –70 m^2^	53	20.6
70–120 m^2^	91	35.4
> 120 m^2^	98	38.1
*Access to outdoor space *^*b*^		
Garden	139	54.1
Terrace	92	35.8
Loggia	106	41.2
No access to outdoor space	37	14.4

*N* = 257

^a^Only one option may be chosen; ^b^More than one may be chosen

#### Interview participants

Semi-structured interviews were conducted with four women and three men, aged 34–65 years. At the time of the interviews, all seven were living in rural or suburban areas. Five were residing in single-family houses with gardens and two in apartments with private outdoor spaces such as terraces. One was living in a multi-person household with school-age children and the others lived in one- or two-person households, either with a spouse or with adult children. Three persons were working full time; two interviewed partners each held part-time jobs, and two were unemployed or had retired.

### Characteristics of home learning environments

In this section of the paper, we present the physical-spatial conditions (including technical equipment) in which the digitally-supported learning of students in academic continuing education took place during the initial COVID-19 restrictions. About 59% of the survey participants carried out activities related to their studies in rooms that were used for purposes other than studying, such as living and leisure activities; 41.2% had their own separate study room; and 25.3% of the respondents had to coordinate the use of their learning place with other people living in the household. Almost three-fourths of the participants (74.7%) had access to their learning place at all times. Office desks were available to 69.6% of participants, while 38.5% studied on dining tables either exclusively or in addition to an office desk. Half had access to an office chair, while 43.2% used a living room chair as work seating either exclusively or additionally. Almost all participants reported having a laptop and/or a desktop computer as available equipment (99.2%). While 6.2% used a desktop computer only, laptops were the electronic devices most frequently used: 93% had a laptop available for studying, and 75.9% used a laptop without an additional desktop computer. About 69.3% used smartphones and 35.4% used tablets in addition to a laptop and/or desktop computer at their learning place. About 1% reported using only a tablet or a tablet in combination with a smartphone for online learning activities. Table 3 illustrates the proportionate availability of office equipment and IT infrastructure elements.

**Table 3:** Physical-spatial conditions of home learning environments

Home learning environment	Physical-spatial conditions	*f*	%
Previous existence of learning place ^b^	Own learning place already available	160	62.3
	Own learning place newly established	38	14.8
	No specially designated learning place available	72	28
	Learning place also used for other purposes	16	6.2
Purpose of the room used for learning ^a^	Dedicated room for studying	106	41.2
	Room also used for other purposes	151	58.8
Location for learning activities ^a^	Predominantly at designated learning place	179	69.6
	Often at other places	78	30.4
Availability of learning place ^a^	Learning place available at all times	192	74.7
	Coordination of learning place with others	65	25.3
Furniture, décor and amenities in the learning place ^b^	Office desk	179	69.6
	Dining or kitchen table	99	38.5
	Office chair	128	49.8
	Armchair	111	43.2
	Desk lamp	138	53.7
	Shelves and storage space	159	61.9
	Images, photos	116	45.1
	Decorative elements	106	41.2
	Indoor plants	106	41.2
	Curtains, carpeting, home textiles	135	52.5
IT equipment ^b^	Laptop	239	93
	Desktop computer	60	23.3
	Docking station	40	15.6
	Tablet	91	35.4
	Smartphone	178	69.3
	Second screen	92	35.8
	External webcam	26	10.1
	External speakers	85	33.1
	Headset	117	45.5
	Printer	175	68.1
	Scanner	139	54.1

*N* = 257

^a^Only one option may be chosen, ^b^more than one may be chosen

The analysis of the interviews yielded similar results. The living situations described in the interviews were all quite spacious, with a learning place either in a separate room or in a designated area. In most cases, the learning place was also used for the home office. In one case, the interviewee had a study room but preferred to conduct online learning activities at the kitchen table in a two-person household with an adult child. Two interviewees had to coordinate the use of their learning place with other household members, while the others did not. While three of the interview participants had ergonomic office furniture in their learning place, the others used ordinary kitchen tables and chairs and, in one case, antique furniture. Regarding technical equipment, all of the interviewees said that they used laptops primarily for distance learning, supplemented at most by a mouse and/or a headset and, in one case, by external speakers. Although several had additional IT equipment such as printers and scanners available, they stated that these were not used for their online learning activities.

### Perception of home learning environment for digitally-supported learning

In this section of the study, we examine how academic continuing education students perceived their home learning environment for digitally-supported learning during the initial COVID-19 restrictions. We investigated 11 attributes of spatial characteristics and indoor environmental conditions, with exploratory factor analysis (EFA) being conducted to discover possible dimensions (underlying factors). The extraction method of principal component analysis and varimax rotation with Kaiser normalisation was used to identify sub-scales that fit together statistically. The EFA produced a two-factor solution based on the varimax rotation with Kaiser normalisation and eigenvalues λ > 1. The two factors explained 55.2% of the variance. The Kaiser–Meyer–Olkin (KMO) measure of sampling adequacy was 0.853 and Bartlett’s Test of Sphericity was significant ($\chi^{2}$ (55) = 1073.03, *p* < 0.001). Table 4 shows the results of the factor analysis with factor loadings.

**Table 4:** Exploratory Factor Analysis (EFA) for spatial characteristics and environmental conditions

Spatial & environmental condition	Factor loading		Degree of communality
	1	2	*h*_*i*_^*2*^
08 distraction-free environment	0.818	0.101	0.68
09 protection against noise pollution	0.777	0.242	0.66
02 ergonomic work-compatible furniture	0.730	0.072	0.54
11 adaptability to individual spatial requirements	0.682	0.375	0.61
01 adequate size	0.613	0.392	0.53
03 appropriate technical equipment	0.503	0.287	0.34
07 good ventilation conditions	0.237	0.795	0.69
04 adequate supply of daylight	0.070	0.708	0.51
06 comfortable temperature conditions	0.262	0.683	0.54
05 pleasant view	0.170	0.668	0.48
10 attractive interior design	0.390	0.603	0.52
Eigenvalues λ	3.21	2.86	6.07
Variance explained (%)	29.2	26.0	55.2

The two factors were interpreted as follows:

- Factor 1: Learning Place Quality (explaining 29.2% of variance)
- Factor 2: Indoor Environmental Quality (IEQ) (explaining 26.0% of variance).

Consistency of perceptual space was verified with Cronbach’s alpha. The values for the reliability coefficient for the two factors ranged from 0.75 to 0.83 (Table 5). Furthermore, for the items of the factors, the item selectivity (*r*_it_) and mean values (*M*) as well as standard deviations (*SD*) were calculated.

**Table 5:** Reliability analysis for factors learning place quality and indoor environmental quality

Factor	Items *k*	*n*	Cronbach α	Selectivity (*r*_*it*_)	*M* (*SD*)
F1 learning place quality	6	248	0.83	0.50–0.69	3.15 (.69)
F2 indoor environmental quality	5	256	0.75	0.48–0.66	3.53 (.51)

1 = disagree, 2 = rather disagree, 3 = rather agree, 4 = agree (that personal requirements were met)

Students were asked about the extent to which their personal requirements were met regarding 11 different attributes. These describe the spatial characteristics and environmental conditions of their predominantly-used learning place and were allocated to one of two identified factors in the exploratory factor analysis (EFA), interpreted as F1 Learning Place Quality and F2 Indoor Environmental Quality. For most of the attributes, students rather agreed (3) or agreed (4) that their requirements were met (Table 6).

**Table 6:** Students’ levels of agreement regarding the fulfilment of their personal requirements for their predominantly-used learning places

*Learning place quality items*	*M*	*SD*
03 appropriate technical equipment	3.51	0.72
01 adequate size	3.41	0.87
09 protection against noise pollution	3.15	0.93
11 adaptability to individual spatial requirements	3.15	0.94
08 distraction-free environment	2.99	1.02
02 ergonomic work-compatible furniture	2.68	1.08
F1 Learning place quality (*n* = 248)	3.15	0.68
*Indoor environment quality items*		
07 good ventilation conditions	3.71	0.54
04 adequate supply of daylight	3.66	0.62
06 comfortable temperature conditions	3.63	0.58
10 attractive interior design	3.39	0.78
05 pleasant view	3.26	0.99
F2 Indoor environmental quality (*n* = 256)	3.53	0.51

1 = disagree, 2 = rather disagree, 3 = rather agree, 4 = agree (that personal requirements were met)

A rather high level of satisfaction (mean ± standard deviation) was found regarding perceived indoor air quality (good ventilation conditions, 3.71 ± 0.54), supply of daylight (3.66 ± 0.62), thermal comfort (comfortable temperature conditions, 3.63 ± 0.58) and technical equipment (3.51 ± 0.72). Only distraction-free environment (2.99 ± 1.02) and ergonomic aspects (ergonomic work-compatible furniture 2.68 ± 1.08) were reported by the students to be less than satisfactory, and there were greater variances in terms of satisfaction.

Interview participants were also satisfied with the spatial characteristics and equipment in the learning place. Sufficient space, quietness, light or brightness and proximity to nature were named as reasons for satisfaction with the living situation as well as with the physical learning environment. Except for the youngest interview partner, who mentioned that he wanted better IT equipment but could not afford it, the interviewees were quite satisfied with their IT equipment. Some expressed astonishment that participation in online learning activities worked well despite their rather basic technical equipment.

### Differences in physical-spatial conditions of home learning environments

We analysed how the physical-spatial conditions of academic continuing education students differed according to gender, age and household structure (with/without children). These factors were chosen based on the literature and our initial exploratory analysis which revealed the factors that are related to differences in equipment and availability of learning place. We present results for the equipment and availability of the learning place.

### Differences in equipment between gender and age groups

#### Gender and equipment

**Fig. 1:**
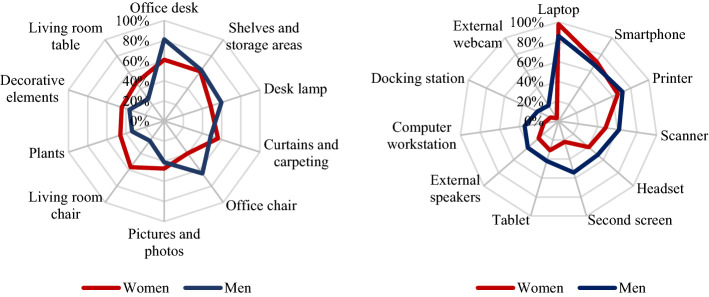
Use of furniture (left) and IT equipment by gender

Of particular interest was understanding whether there were any differences in how students’ learning places were equipped and what kinds of furniture and technology were predominantly available according to gender. Figure 1 presents the percentages of ownership of identified furniture and technical equipment.

The results of chi-square ($\chi^{2}$) tests indicated that significant differences existed between men and women regarding the use of the following items of furniture: office desk, $\chi^{2}$ (1, 253) = 12.077, *p* = 0.001; dining/kitchen table, $\chi^{2}$ (1, 253) = 10.572, *p* = 0.001; office chair, $\chi^{2}$ (1, 253) = 15.142, *p* < 0.001; ordinary chairs, $\chi^{2}$ (1, 253) = 26.679, *p* < 0.001; and indoor plants, $\chi^{2}$ (1, 253) = 3.836, *p* = 0.05. There were no significant differences regarding desk lamps, shelves and storage, pictures/photos, decorative elements and curtains/carpeting. The striking result here is that men’s learning places were more often equipped with office desks (81.3%) and office chairs (64.5%) compared with women’s places. Women used mainly dining or kitchen tables (47.3%), while only 27.1% of the men used dining or kitchen tables. Similarly, a majority of the women (56.8%) used ordinary chairs rather than office chairs in their learning place.

Regarding technology, significant differences were found between men and women in the adoption and use of some categories of equipment, including laptops, $\chi^{2}$ (1, 253) = 13.374, *p* < 0.001; personal computers, $\chi^{2}$ (1, 253) = 13.145, *p* < 0.001; docking stations, $\chi^{2}$ (1, 253) = 10.116, *p* = 0.001; second screens, $\chi^{2}$ (1, 253) = 28.087, *p* < 0.001; external webcams, $\chi^{2}$ (1, 253) = 16.162, *p* < 0.001; speakers, $\chi^{2}$ (1, 253) = 5.816, *p* = 0.016; and scanners, $\chi^{2}$ (1, 253) = 4.687, *p* = 0.030. No significant differences were found concerning technical equipment such as tablets, smartphones, headsets and printers.

Similar to the situation regarding learning place furnishings, men were found to be better equipped technically compared with women. The data show that men, in particular, used IT equipment more often than women did, which can be characterised as going beyond the fundamentally necessary basic infrastructure to participate in online learning (e.g. external webcams and speakers, second screens).

#### Gender and satisfaction with equipment

Mann–Whitney U tests were used to analyse whether there was a significant difference between men’s and women’s perceptions of whether their personal requirements had been met in regard to *ergonomic work-compatible furniture* as well as *appropriate technical equipment*.

Our results revealed a small but significant difference in the perception of fulfilled personal requirements regarding *ergonomic work-compatible furniture*, *U*(*N*_female_ = 145, *N*_male_ = 107) = 6205.00, *z* = -2.81, *p* = 0.005, *r* = -0.18 (small effect). More women considered their personal requirements regarding ergonomic furniture as not having been satisfied. Concerning the *perception of fulfilled personal requirements regarding appropriate technical equipment,* no significant difference was found between female and male students, *U*(*N*_female_ = 146, *N*_male_ = 106) = 7587.00, *z* = -0.310, *p* = 0.757, *r* = -0.02.

These results indicate that female participants were less satisfied with the ergonomic furniture in their learning place, but there was no significant difference regarding satisfaction with technical equipment, although the specified equipment differed between men and women.

The interview results did not point to a clear difference between men and women. Still, one female participant mentioned that she prefers to manage without ergonomic furniture and IT equipment such as a second screen, scanner or printer, which are not necessary for learning, because of the aesthetic appearance of the learning environment. Another woman noted that she prefers to work exclusively with her laptop (“I love this thing”) even if supplementary equipment (e.g. second screen, external keyboard) is available.

#### Age and equipment

The next factor investigated was differences in equipment (furnishings and technology) according to age (five categories: 24 years and younger, 25–34, 35–44, 45–54 and 55 + years). Differences were tested using $\chi^{2}$ tests. Significant differences were identified regarding furnishings between the age categories.

The analysis showed a continuous increase in the use of desk lamps with age, $\chi^{2}$ (1, 255) = 10.388, *p* = 0.034. With regard to shelves and storage, $\chi^{2}$ (1, 255) = 11.736, *p* = 0.019; pictures/photos, $\chi^{2}$ (1, 255) = 12.531, *p* = 0.014; decorative elements, $\chi^{2}$ (1, 255) = 10.997, *p* = 0.027; and indoor plants $\chi^{2}$ (1, 255) = 15.859, *p* = 0.003, higher values were found in the younger- and older-age categories compared with the middle-age category. No significant differences were found concerning furnishings such as an office desk, using a dining/kitchen table, an office chair, using an ordinary chair and curtains/carpeting.

Furthermore, differences in the technical equipment in the learning place according to age categories were investigated. Interestingly, a significant difference between the age categories was found only for printers. Learning places were more likely to be equipped with a printer with the increasing age of the user ($\chi^{2}$ (1, 255) = 10.043, *p* = 0.040).

No significant differences were found concerning technological equipment such as a laptop, personal computer, docking station, tablet, smartphone, second screen, external webcam, speakers, headset and scanner.

### Differences in the availability of a learning place between gender and household structure

In this section, we examine the role of gender and household structure in the availability and purpose of the learning place.

#### Gender and availability of learning place

The following characteristics of the predominantly-used learning places of the participants were examined: *purpose of the room used for learning, fixed location for learning activities* and *availability of the learning place*. $\chi^{2}$ tests were conducted to determine the differences between the groups.

Significant differences were found regarding the purpose of the room (if it was used only for studying) and whether a dedicated place for learning was available. Fewer female participants reported having access to a separate study room, $\chi^{2}$ (1, 253) = 5.285, *p* = 0.022, while male students reported conducting online learning activities more often in their own study room and at a designated learning place, $\chi^{2}$ (1, 253) = 4.378, *p* = 0.036. No significant differences between men and women were found regarding the temporal availability of the learning place.

#### Household structure (with or without children) and availability of learning place

Regarding the characteristics of the learning environment, *purpose of the room used for learning*, c*hanging location for learning activities,* and *availability of the learning place* were analysed according to the household structure (whether children were living in the household or not) with a *χ*^2^ test.

No significant differences were found between participants in households with and without children regarding the purpose of the room used for learning and the changing of location for learning activities. However, there was a significant difference regarding the temporal availability of the learning place based on household structure, $\chi^{2}$ (1, 253) = 12.944, *p* < 0.001. In households with children, the use of the learning place had to be coordinated with others more often than in households without children.

The relationship between the physical-spatial conditions in the home learning environment and participants’ well-being and learning experience.

In the final section of the study, we investigated the role of different spatial characteristics on well-being and learning experience.

#### Perceived influence of physical learning environment on learning experience

We wanted to know how participants perceived the impact of the physical learning environment on learning experience regarding motivation, concentration, learning performance and well-being at the learning place. These factors were examined with five items using a 7-point rating scale. Table 7 shows descriptive data (mean values [*M*] and standard deviations [*SD*]) for the questionnaire items on learning experiences during the initial COVID-19 restrictions.

**Table 7:** Mean values (*M*) and standard deviations (*SD*) for the items of perceived impact of learning environment on learning experience

Learning experience (*n* = 257)	*M (SD)*
*Perceived influence of physical learning environment on*	
Motivation to learn (negative–positive)	0.85 (1.71)
Concentration (negative–positive)	0.65 (1.76)
Learning performance (negative–positive)	0.81 (1.61)
Well-being at learning place (unwell–well)	1.72 (1.37)
Perceived suitability of learning place (unsuitable–suitable)	1.39 (1.67)

Negative – 3, – 2, – 1, 0, 1, 2, 3 positive; unwell – 3, – 2, – 1, 0, 1, 2, 3 well; unsuitable – 3, – 2, – 1, 0, 1, 2, 3 suitable

On average, the influence of the physical learning environment on motivation, concentration and learning performance was perceived as neutral to slightly positive. Well-being at the learning place and suitability were rated rather positively.

In the interviews, perceptions in connection with the physical learning environment were mentioned, particularly when they were related to problems and dissatisfaction. For example, noise pollution, non-permanent availability of the learning place, non-ergonomic furniture and/or insufficient IT equipment were identified as factors influencing concentration. The possibility of self-determined and unobserved behaviours during online learning activities, such as getting up, moving around, or fetching coffee during online courses, were considered beneficial compared with face-to-face lectures but, at the same time, it was seen as an impediment to concentration. Participants did not make a connection between learning place and learning experience: “No, I see that totally disconnected, it has no influence, neither positive nor negative.” Another participant indicated that “I wouldn’t say that the motivation to learn depends on the place of learning – no, that’s in my head”. However, an influence of the spatial learning situation on concentration was confirmed with comments such as the following: “When I have peace and can be alone, that has the greatest influence on my concentration” and “I am undisturbed in this room. That means I can concentrate there quite easily, which of course has an influence”.

#### Room purpose and perceived influence of physical learning environment on learning experience

We also investigated the purpose of the room predominantly used for learning activities in terms of the availability of one’s own study room or the multi-purpose use of the main room for learning. We examined how the availability of the room or its multi-purpose use affected the perceived influence of the learning environment on students’ learning experiences. Independent *t*-tests were used to determine whether there was a statistically-significant difference between the two groups (Table 8) shows the results.

**Table 8:** Room purpose and perceived influence of physical learning environment on learning experience

Learning experience	Purpose of the room used for learning				*t* (df)*, p*	Cohen’s *d*
	Own study room		Room also used for other purposes			
	*n*	*M (SD)*	*n*	*M (SD)*		
*Perceived influence of physical learning environment on*						
Motivation to learn (negative–positive)	106	1.42 (1.42)	151	0.46 (1.79)	4.78 (251.07), < .001	.58
Concentration (negative–positive)	106	1.37 (1.50)	151	0.15 (1.77)	5.94 (245.72), < .001	.73
Learning performance (negative–positive)	106	1.39 (1.38)	151	0.41 (1.64)	5.17 (246.76), < .001	.64
Well-being at learning place (unwell–well)	106	2.08 (1.13)	151	1.46 (1.46)	3.78 (252.79), < .001	.46
Suitability of learning place (unsuitable–suitable)	106	2.16 (1.15)	151	0.85 (1.77)	7.17 (253.52), < .001	.85

Negative – 3, – 2, – 1, 0, 1, 2, 3 positive; unwell -– 3, – 2, – 1, 0, 1, 2, 3 well; unsuitable – 3, – 2, – 1, 0, 1, 2, 3 suitable

As shown in Table 8, all comparisons were significant with medium and high effect sizes (Cohen’s *d*). Students who predominantly used a separate study room stated more frequently that the physical environment has a positive influence on their learning experiences (motivation, concentration, learning performance) than students whose learning place was also used for other purposes such as dining. Furthermore, students with their own study room felt more comfortable and rated it as more suitable than the comparison group.

#### Fixed or changing location for learning activities and perceived influence of physical environment on learning experience

We further used a *t*-test to analyse the role of having a fixed or alternating location for learning activities (whether the activities were conducted predominantly at one designated learning place or often at other places) regarding the perceived influence of the learning environment on the students’ learning experiences.

Table 9 shows that all differences between the two groups were significant with medium and high effect sizes (Cohen’s *d*). Students whose learning activities were conducted predominantly at a fixed learning place stated more frequently that the physical environment had a positive influence on their learning experiences (motivation, concentration, learning performance) than students who often carried out their learning activities in other places. Moreover, students who studied in a designated learning place reported feeling more comfortable and rated it as more suitable than the comparison group.

**Table 9:** Fixed or changing location for learning activities and perceived influence of physical learning environment on learning experience

Learning experience	Location for learning activities				*t* (df), *p*	Cohen’s *d*
	Predominantly at the designated learning place		Often at other places			
	*n*	*M* (*SD*)	*n*	*M* (*SD*)		
*Perceived influence of physical learning environment on*						
Motivation to learn (negative–positive)	179	1.06 (1.60)	78	0.37 (1.86)	2.85 (128.97), .005	.41
Concentration (negative–positive)	179	0.85 (1.71)	78	0.21 (1.80)	2.67 (140.08), .008	.37
Learning performance (negative–positive)	179	1.01 (1.53)	78	0.36 (1.70)	2.92 (133.79), .004	.41
Well-being at learning place (unwell–well)	179	1.91 (1.19)	78	1.27 (1.63)	3.13 (113.89), .002	.48
Suitability of learning place (unsuitable–suitable)	179	1.68 (1.50)	78	0.74 (1.87)	3.90 (122.00), < .001	.58

Negative – 3, – 2, – 1, 0, 1, 2, 3 positive; unwell – 3, – 2, – 1, 0, 1, 2, 3 well; unsuitable – 3, – 2, – 1, 0, 1, 2, 3 suitable

The interviews also make clear that, for some students, a fixed place and certain spatial environment is important for creating an atmosphere conducive to learning. One interviewee stated: “All kinds of places in my home can be used for learning, but it is important for me to have a fixed learning place to get into the right mood.” For others, having the possibility to change the learning place, for example, to an outdoor space, was seen as an advantage of online learning compared with face-to-face lectures.

#### Availability of learning place and perceived influence of physical environment on learning experience

We also investigated the impact of having a learning place that was available at all times (in contrast to having to coordinate use with other persons) on the perceived influence of the physical learning environment on the students’ learning experiences. Independent *t*-tests were used to determine the difference between the two groups.

Table 10 shows that all differences between the two groups were significant, with medium and high effect sizes (Cohen’s *d*). Students whose learning place was available at all times more often stated that the physical learning environment had a positive influence on their learning experience (motivation, concentration, learning performance) than students whose learning place was also used by other persons. In addition, students whose learning place was available at all times felt more comfortable and rated it as more suitable than the comparison group.

**Table 10:** Availability of learning place and perceived influence of physical learning environment on learning experience

Learning experience	Availability of the learning place				*t* (df), *p*	Cohen's *d*
	Available at all times		Use had to be coordinated with others			
	*n*	*M* (*SD*)	*n*	*M* (*SD*)		
*Perceived influence of physical learning environment on*						
Motivation to learn (negative–positive)	192	1.09 (1.60)	65	0.14 (1.84)	3.74 (98.90), < .001	.58
Concentration (negative–positive)	192	0.94 (1.62)	65	-.20 (1.91)	4.33 (97.14), < .001	.67
Learning performance (negative–positive)	192	1.06 (1.51)	65	0.08 (1.66)	4.23 (102.29), < .001	.64
Well-being at learning place (unwell–well)	192	1.96 (1.21)	65	1.00 (1.54)	4.56 (92.37), < .001	.74
Suitability of learning place (unsuitable – suitable)	192	1.69 (1.51)	65	0.51 (1.81)	4.74 (95.94), < .001	.74

Negative – 3, – 2, – 1, 0, 1, 2, 3 positive; unwell – 3, – 2, – 1, 0, 1, 2, 3 well; unsuitable – 3, – 2, – 1, 0, 1, 2, 3 suitable

### Influence of physical home learning environment on motivation, stress and well-being

Our data revealed that the average well-being score measured by the WHO Well-Being Scale was 15.02, where 0 is the worst possible well-being and 25 is the best possible well-being (13 and below is considered poor well-being). Clearly, our participants did not have the best possible well-being during the first lockdown due to the COVID-19 pandemic. On the other hand, the average stress level measured by the PSQ was reported to be 37.5, where scores range from 0 to 100 and a higher score indicates a higher reported stress level. Worry was reported to be even lower than general stress level (mean score was 28.4) during the first lockdown in Austria. Motivation to learn was reported to be high (*M* = 3.99). It can be summarised that our participants were not stressed and were motivated to learn during the initial COVID-19 restrictions, even though they did not feel perfectly fine. A noteworthy result that emerged from the interviews was the lack of personal contact. All interview participants, especially those in their first semester, were concerned about not being able to come together with their classmates and the lecturers, and they reported a negative impact of the lack of personal contact on their well-being, even if the elimination of travel time to the campus was seen as an advantage.

Three hierarchical multiple regression analyses were performed to determine how personal factors and physical-spatial conditions influenced students’ motivation, stress and well-being. Mean scores of the standardised questionnaires (motivation: LEIMO; stress: PSQ; well-being: WHO 5) were calculated and served as the criteria variables. Predictors were determined as students’ gender, which was a dichotomous variable (male or female); age (continuous variable with minimum = 22 years, maximum = 65 years, *M* = 40.46, *SD* = 9.21); children in the household (dichotomous variable: yes, no); Learning Place Quality (calculated mean score); Indoor Environmental Quality (calculated mean score); availability of the learning place at all times (dichotomous variable: yes, no); and previous experience with online learning (dichotomous variable: yes, no). In order to determine whether the regression model could be generalised, we considered that the underlying assumptions had been fulfilled, such as intercorrelation among predictor variables, multicollinearity, normal distribution of residual errors and autocorrelations, by calculating the Durbin-Watson coefficient. The results show that no high levels of intercorrelation between predictor variables could be found, and that none of the correlations between predictor variables exceeded the critical value of 0.80 for multicollinearity. The normal distributions of residual errors were confirmed (normal curve of histogram; normal probability represents an approximately 45-degree line). The data met the assumption of independence of observations because the Durbin-Watson coefficient (*d*) was between 1.5 and 2.5 (motivation: *d* = 1.922, stress: *d* = 2.037, well-being: *d* = 2.035).

Because all assumptions were met, hierarchical regression analysis was conducted. The predictors were entered into regression as two blocks. In the first step, gender, age and having children living in the household were entered to control their effects; in the second step, Learning Place Quality, Indoor Environmental Quality and temporal availability of the learning place were entered into the regression analyses. Table 11 shows the results for each model.

**Table 11:** Results of regression models of predictors of motivation, stress and well-being

Predictor	Motivation (*n* = 245)		
	*b*	*SE b*	β
Step 1 *(R* = .105, *R*^2^ = 1.1%, *R*^2^_adj_ = − 0.1%)			
Constant	4.02	.26	
Gender (female; male)	– .14	0.09	− 0.09
Age (years)	< 0.01	< 0.01	0.04
Children in the household (1 = no; 0 = yes)	0.07	0.10	0.05
Step 2 *(R* = 0.258*, R*^2^ = 6.7%, *R*^2^_adj_ = 3.9%)			
Constant	3.38	0.48	
Gender (female; male)	– 0.17	0.10	− 0.11
Age (years)	< 0.01	0.01	< − 0.01
Children in the household (1 = no; 0 = yes)	0.03	0.10	0.02
F1 Learning Place Quality (score)	0.27	0.09	0.25^**^
F2 Indoor Environmental Quality (score)	– 0.01	0.12	− 0.01
Availability of learning place at all times (yes; no)	0.03	0.12	0.02
Previous experience with online learning (yes; no)	0.03	0.10	0.02

	Stress (*n* = 248)		
	*b*	*SE b*	β
Step 1 (*R* = 0.225, *R*^2^ = 5.1%, *R*^2^_adj_ = 3.9%)			
Constant	49.73	7.16	
Gender (female; male)	3.86	2.65	0.09
Age (years)	– 0.35	0.15	− 0.15^*^
Children in the household (1 = no; 0 = yes)	– 7.47	2.69	− 0.18^**^
Step 2 (*R* = 0.394, *R*^2^ = 15.9%, *R*^2^_adj_ = 13.4%)			
Constant	79.45	12.99	
Gender (female; male)	3.44	2.60	0.08
Age (years)	– 0.18	0.14	− 0.08
Children in the household (1 = no; 0 = yes)	– 5.76	2.66	− 0.14^*^
F1 Learning Place Quality (score)	– 4.27	2.48	− 0.14
F2 Indoor Environmental Quality (score)	– 7.96	3.12	− 0.19^*^
Availability of learning place at all times (yes; no)	4.57	3.28	0.09
Previous experience with online learning (yes; no)	– 2.43	2.61	− 0.06

	Well-being *(n* = 246)		
	*b*	*SE b*	β
Step 1 (*R* = 0.137, *R*^2^ = 1.9%, *R*^2^_adj_ = 0.7%)			
Constant	2.65	0.37	
Gender (female; male)	– 0.18	0.14	− 0.08
Age (years)	0.01	0.01	0.11
Children in the household (1 = no; 0 = yes)	0.15	0.14	0.07
Step 2 (*R* = 0.370, *R*^2^ = 13.7%, *R*^2^_adj_ = 11.1%)			
Constant	1.08	0.68	
Gender (female; male)	– 0.15	0.14	− 0.07
Age (years)	< 0.01	0.01	0.04
Children in the household (1 = no; 0 = yes)	0.06	0.14	0.03
F1 Learning Place Quality (score)	0.28	0.13	0.18^*^
F2 Indoor Environmental Quality (score)	0.38	0.16	0.18^*^
Availability of learning place at all times (yes; no)	– 0.21	0.17	− 0.09
Previous experience with online learning (yes; no)	– 0.03	0.14	− 0.01

Criteria = motivation, stress, well-being; Predictor Step 1 = gender, age, children; Predictor Step 2 = factor 1 ‘Learning Place Quality’; factor 2 ‘Indoor Environment Quality’; availability of learning place at all times, previous experience with online learning

^**^p < 0.01, *p < 0.05

Regression analysis for motivation shows that the multiple correlation coefficient between the linear combination of three predictors (i.e. gender, age and children living in the household) and motivation was *R* = 0.105. Model 1 was not significant, *F*(3,241) = 0.90, *p* = 0.444, *R*^2^ = 0.011, *R*^2^_adj_ = -0.001. In Model 2, the multiple correlation coefficient between the linear combination of four predictors (i.e. the combination of Learning Place Quality, Indoor Environmental Quality, availability of learning place at all times and previous experience with online learning and motivation) increased to *R* = 0.258 after controlling for the effects of the demographic statistics gender, age and children living in the household. Model 2 significantly predicted motivation, *F*(7,237) = 2.42, *p* = 0.021, *R*^2^ = 0.067, *R*^2^_adj_ = 0.039. The combined factors of Learning Place Quality, Indoor Environmental Quality, availability of learning place and previous experience with online learning accounted for 3.9% of the variance in motivation above the demographic characteristics. According to standardised coefficients (β), there was a positive relationship between Learning Place Quality and motivation. In this model, only Learning Place Quality had an impact on motivation (β = 0.25).

Regarding the prediction of stress, the multiple correlation coefficient between the linear combination of three predictors (i.e. gender, age and children in the household) was *R* = 0.225. Model 1 significantly predicted stress perception, *F*(3,244) = 4.33, *p* = 0.005, *R*^2^ = 0.051, *R*^2^_adj_ = 0.039. The combination of these three predictors accounted for 3.9% of the variation in stress perception. According to standardised coefficients (β), there was a negative relationship between age and children and stress, and having children living in the household and lower age have an impact on increasing students’ stress perception. Statements from the interviews refer to the importance of age in coping with stressful situations. An older, retired person noted being more relaxed in stressful situations and expressed understanding and empathy towards younger colleagues, especially if they had to take care of children.

In Model 2, the multiple correlation coefficient between the linear combination of four predictors (i.e. the combination of Learning Place Quality, Indoor Environmental Quality, availability of the learning place at all times and previous experience with online learning) and stress increased to *R* = 0.394 after controlling for the effects of the demographic statistics of gender, age and children in the household. Model 2 significantly predicted stress, *F*(7,240) = 6.46, *p* < 0.001, *R*^2^ = 0.159, *R*^2^_adj_ = 0.134. The combined factors of Learning Place Quality, Indoor Environmental Quality, availability of learning place and previous experience with online learning accounted for 13.4% of the variance in stress above the demographic characteristics. According to standardised coefficients (β), there was a negative relationship between having children in the household and Indoor Environmental Quality and stress; having children increases stress (β = -0.14) as does Indoor Environmental Quality (β = -0.19).

Furthermore, regression analysis for well-being showed a multiple correlation coefficient of *R* = 0.137 between the linear combination of three predictors (i.e. gender, age and children) and well-being. Model 1 was not significant, *F*(3,242) = 1.55, *p* = 0.202, *R*^2^ = 0.019, *R*^2^_adj_ = 0.007. In Model 2, the multiple correlation coefficient between the linear combination of four predictors (i.e. the combination of Learning Place Quality, Indoor Environmental Quality, availability of learning place, and previous experience with online learning) and well-being increased to *R* = 0.370 after controlling for the effects of the demographic statistics of gender, age and children. Model 2 significantly predicted well-being, *F*(7238) = 5.39, *p* < 0.001, *R*^2^ = 0.137, *R*^2^_adj_ = 0.111. The combined factors of Learning Place Quality, Indoor Environmental Quality, and availability of learning place accounted for 11.1% of the variance in well-being above the demographic characteristics. According to standardised coefficients (β), there was a positive relationship between Learning Place Quality (β = 0.18) and Indoor Environmental Quality (β = 0.18), which had an impact on well-being.

In this research, how well spatial characteristics such as Learning Place Quality, Indoor Environmental Quality, the continuous availability of the learning place, and previous experience with online learning predict motivation, stress, and well-being were explored. Variables such as gender, age and children living in the household were considered as demographic characteristics as well. According to the results of the analyses, spatial characteristics explained 3.9% variance in motivation, 13.4% in stress, and 11.1% in well-being after controlling for gender, age and children. It is important to emphasise that Learning Place Quality had a higher impact on motivation (β = 0.25) than on well-being (β = 0.18). The influence of Indoor Environmental Quality on stress (β = -0.19) and on well-being (β = 0.18) showed a very similar impact.

To summarise, the quality of the physical learning environment had an impact on motivation and well-being, and the lack of indoor environmental quality increased stress while its availability led to well-being. Having children living in the household appeared to increase stress, which also appeared to decrease with older age. Gender, all-time availability of the learning place and previous experience with online learning did not have any significant effects on motivation, stress and well-being.

## Discussion

We discuss the key findings in four main parts following the research questions. The first part concerns the physical-spatial conditions of students in academic continuing education (including technical equipment) in which digitally-supported learning took place during the initial COVID-19 restrictions. The academic continuing education students participating in our study were ‘quite well equipped’ at the beginning of the lockdown; many already had a workplace (with an office desk, chair and printer) that was available for them all the time (i.e. they didn’t have to coordinate with someone else); they were living in large flats; and about one-third were living in households that included children whose age required care (preschool or compulsory school age). In general, they had access to the IT equipment necessary to participate actively in digitally-supported learning settings. The observable differences potentially impacted the quality of participation (e.g. better sound quality, larger screens, etc.) but not the fundamental opportunity to participate. In that sense, we did not observe a ‘digital divide’ in our population with respect to available IT infrastructure, as described by DiMaggio et al. (2004). This can be explained by the economic situation of the observed student population who, because of their ages and career advancement, usually pursued their studies in an economically more-stable and settled environment than traditional students. Juxtaposing this finding on the conditions of traditional students manifests the different living conditions of the traditional and non-traditional student groups. More than half of the students were living in single or multi-person households without children, and more students’ living areas were larger than 120 m^2^. In 2020, the average living space of Austrian households was 99.9 m^2^, while the average living space per person was 45.5 m^2^ (Statistik Austria, 2021). The EUROSTUDENT study (Hauschildt et al., 2018) highlights university students’ living conditions: they live mainly in their parents’ home (36%), student housing (18%) or in flats shared with others (15%).

Our findings concerning the physical-spatial conditions of academic continuing education students suggest that, while most of the participants reported quite spacious living conditions that often allowed for dedicated learning places, the blurred boundaries (Ahrentzen, 1990) between the different life domains confronted by the population targeted in this study often impacted their learning environment. In particular, more often than traditional students, continuing education students have to balance work, family and studies and are confronted with competing interests regarding workspace occupation by other members of the household. This becomes particularly visible in the data on learning place availability for students with children, who had to coordinate the availability of the physical space for learning activities with others significantly more often than the rest of the population. This finding is in line with previous research (e.g. Panacci, 2015) suggesting that the struggle to manage competing roles (e.g. parenting, caregiving, employment and community involvement) has also been described as a major challenge for mature students.

For the second part, we delved into how our participants perceived their home learning environment for digitally-supported learning during the initial COVID-19 restrictions in terms of noise, comfort, aesthetic, air quality and other indoor environment qualities. Our analysis suggests that continuing education students perceived their home learning environment as being mostly adequate, which contradicts the findings of studies conducted with traditional students revealing that reported a lack of learning space, a favourable learning environment, technical equipment and internet access as barriers to participation in online learning during the COVID-19 lockdowns (Baticulon et al., 2021; Kapasia et al., 2020). While the majority of students in our study also reported that their requirements regarding a comfortable, quiet and distraction-free learning environment were met, Baticulon et al. (2021) underlined that, in their sample, “having a quiet study area was a privilege for majority of the students” (p. 6). Thus, we can infer that continuing education students have more favourable home learning environments compared with traditional university students.

The third theme that we want to address involves differences in the physical-spatial learning conditions according to gender, age and household structure (with/without children). Regarding gender, our research identified a significant difference in the technical equipment used by men and women, which aligns with previous studies reporting a significant and “persistent gender gap in access and use of digital technologies” (Davaki, 2018, p. 10). In addition to differences in equipment, we observed differences in the location where the participants were engaging in online learning activities. Fewer female students had access to a separate room and ergonomic furniture for studying. Men tended to engage in online learning activities more frequently in workspaces set up exclusively for learning or working, while women were more likely to use locations or spaces that were used for other purposes besides online learning activities. Research has shown that the digital gap is closely related to socioeconomic inequality (Cruz-Jesus et al., 2016). However, as we do not have data about the SES levels of the participants, we cannot draw conclusions about the reasons for this gap in our sample. On the other hand, a striking finding is the lack of difference between men and women in the perceived overall suitability of their learning environments. Although their learning environments differed significantly according to equipment, ergonomic furniture, and availability of a dedicated place for learning, women rated their learning environments as sufficient for their needs. Existing studies of gender differences in occupants’ satisfaction with indoor environmental quality also identified differences in satisfaction levels regarding specific factors while, at the same time, there were no differences in overall satisfaction with the indoor environment (Kim et al., 2013; S. Lee et al., 2018). Having children in the household, by contrast, played a more important role in having a separate place for learning where students with children often have to coordinate the use of the learning place. Regarding age, our study did not demonstrate significant differences in the physical-spatial conditions of the learning place of students according to their ages.

In the last part, we explored the influence of different physical-spatial conditions on the well-being and learning experience of participants. It was evident that having a separate study room positively influenced the learning experiences of motivation, concentration and learning performance, as well as contributing to students’ well-being in the learning place. Moreover, if the space was used exclusively for learning or working, and if it was available to students at all times (requiring no coordination with others), students perceived the influence of the physical learning environment on motivation, concentration and learning performance more positively and felt more comfortable in their learning environment.

In the model that we tested, we examined the role of different physical-spatial conditions (indoor environmental quality, learning place quality, availability of a fixed learning place and previous experience with online learning) on mental well-being, stress and motivation for learning. Consistent with previous studies (Han et al., 2019; Jamaludin et al., 2016; L. Xiong et al., 2018), we observed that quality of learning place significantly influences motivation for learning, while indoor environment quality did not emerge as a significant predictor. Moreover, in terms of well-being, parallel to evidence previously established (Choi et al., 2014; Clark et al., 2007; Codinhoto et al., 2009; Mujan et al., 2019), both indoor environment quality and quality of the learning place had a small but significant impact on the mental well-being of continuing education students during the COVID-19 pandemic. Regarding stress, contrary to previous research on stress (Rossi et al., 2020; Y. Wang et al., 2020; J. Xiong et al., 2020), the stress levels of our students did not differ according to gender but, as these studies indicated, age played a significant role; in our case, younger students also reported a higher level of stress.

The impact of having children living in the household was observed only for stress. Research (Kossek et al., 2021; Panacci, 2015) investigating blurring boundaries between work, family and studies underline the burden of parenting, especially when the children are young. A current study by Yildirim and Eslen-Ziya (2021) showed that having children appears to be one of the most important predictors of the perceived effect of the pandemic, and the gender gap becomes significant for women academics with children. Kossek et al. (2021) highlighted that having children has a greater impact on women’s well-being. Another recent study by Mayer (2020) presented the connection between children and stress during the initial COVID-19 restrictions. Thus, our results are in line with the findings of previous research and underline the importance of providing students, particularly those who pursue their studies in a non-traditional setting, with opportunities to participate in digitally-supported learning activities that they can adapt to their needs and individual study conditions, including the physical-spatial environment.

With this study, we aimed to delve into two under-researched areas in learning environment research: the physical learning environment for online learning and the learning environment in academic continuing education. While the COVID-19 pandemic has created a setting in which these two topics could be explored on a large scale, the relevance of the findings is not constrained to this particular situation. Future directions for higher education and academic continuing education point to more flexible and blended or online learning provisions (Pelletier et al., 2021; Schulte et al., 2020) in line with students’ demands for alternative and more-flexible learning opportunities (Valtonen et al., 2021) for which the home learning environment as well as other informal learning environments gain significance and importance. Our research also contributes to understanding the relationship between physical, psychological, technological and cultural aspects of learning environments, especially from the perspective of designing student-centred learning environments (Land & Hannafin, 2012) not only for non-traditional students but also for traditional students. The identified effects on motivation and well-being, as well as potential differences moderated by gender, age and household structure, have practical implications and lead to a number of potentially relevant aspects to be further examined in future research. From the perspective of Radcliff’s (2009) *Pedagogy, Space, Technology* (PST) framework, our findings open up new discussions regarding the application of this framework in the physical conditions of informal learning environments used especially for online/distance learning. From a practical perspective, the results can inform the design of individual learning environments at home when engaging in online learning activities and should also be considered from an institutional and pedagogical perspective when conceptualising and designing remote/online learning programmes. From a research perspective, the current study makes evident the need to explicitly consider the role of physical learning environments not only for learning activities in formal educational settings, but also in online settings, especially concerning the heterogeneous conditions that shape learners’ needs and opportunities for participation.

### Limitations

The limitations of the present study include methodological constraints. Our study was subject to selection bias both for the survey and the interviews. For the survey, we did not follow a random sampling approach. For the interviews, interviewees volunteered to participate, which might suggest that people with favourable conditions in terms of available time resources, stress load, etc. were more likely to take part. Another limitation is self-reporting bias. Our results are based on the statements of the participants regarding their learning environment. In particular, one could argue that the fact that the examined learning environments were created by the participants themselves might lead to higher satisfaction, well-being and motivation than could be expected when participants are confronted with learning environments that might be less tailored to their needs (e.g. due to socioeconomic constraints). The authors are currently conducting a follow-up study with participants with a less-favourable socioeconomic background to examine this potential bias more closely. Lastly, our study was limited to how students perceived their home learning environment and their motivation, stress and well-being during the initial COVID-19 restrictions in Austria.

### Further research suggestions

Looking forward, we want to suggest a number of directions for future research. Our study focussed only on academic continuing education students and their physical home learning environment. In order to investigate the differences and build evidence for comparisons between students in higher education and academic continuing education, a study focussing on both groups is strongly suggested. Differences between students in different academic fields were not investigated in this study, although their requirements regarding the physical spatial environment including technical equipment for digitally-supported learning might differ; this also could be of interest for future research. Gender emerged as an issue in our study, but another research area deserving attention would focus on the needs, expectations and satisfaction regarding the learning environment and the impact of the physical spatial conditions of the learning environment. Lastly, we did not include SES as a factor in our study and models; however, based on the existing evidence on its impact, especially for the digital divide, future studies should certainly inquire about the role of SES.

## Conclusions

In this study, we aimed to examine and understand the physical home learning environments of adult learners studying at a continuing education university and their relationship to digitally-supported learning during the initial COVID-19 restrictions. We have described the physical-spatial conditions for digitally-supported learning and analysed how the physical home learning environment impacts adult continuing education students’ mental well-being and learning experiences. To the best of our knowledge, this is the first study of this topic, and thus it contributes to understanding of the effects of spatial features of physical learning environments on remote online-learning activities. Our findings indicated that the examined group of students involved generally have spacious living conditions and almost all the equipment needed for digitally-supported learning. Regarding gender and household structure, significant differences were found in technical equipment, ergonomic furniture and availability of a fixed place for learning. During the restrictions and their learning sessions, the participants reported a low level of stress and positive well-being in general. The greater extent to which students perceived that their learning place (regarding learning place quality and indoor environmental quality) met their needs, the higher were their motivation and well-being and the lower was their stress. Additionally, students who had their own separate and fixed place that did not require coordination with others had better learning experiences. Based on our key findings and existing evidence, we want to quote the eminent work of Virginia Woolf, which still resonates today, and apply it to learning space. “A woman must have money and a room of her own if she is to write fiction” rephrased as “A student must have a room of her or his own and the necessary equipment to have better learning outcomes and mental well-being.”
