# Correction to: Physical home learning environments for digitally‑supported learning in academic continuing education during COVID‑19 pandemic

**DOI:** 10.1007/s10984-022-09412-2

**Published:** 2022-06-22

**Authors:** Filiz Keser Aschenberger, Gregor Radinger, Sonja Brachtl, Christina Ipser, Stefan Oppl

**Affiliations:** 1 Donau-Universität Krems, Krems, Austria; 2 Department for Continuing Education Research and Educational Technologies, Danube University Krems, Dr.-Karl-Dorrek-Straße 30, 3500 Krems, Austria; 3 Department for Building and Environment, Danube University Krems, Dr.-Karl-Dorrek-Straße 30, 3500 Krems, Austria

## Correction to: Learning Environments Research https://doi.org/10.1007/s10984-022-09406-0

In the original publication of the article, some of the paragraphs under the sections “Characteristics of home learning environments’’ and “Perception of home learning environment for digitally-supported learning” were unknowingly deleted by mistake. The complete sections are provided in this correction.

Hence, Table 4 and 5 citations are missed in the article. Also, Tables 4 to 11 were wrongly located, and table 6 header correction also missed.

The original article has been corrected.

### Characteristics of home learning environments

In this section of the paper, we present the physical-spatial conditions (including technical equipment) in which the digitally-supported learning of students in academic continuing education took place during the initial COVID-19 restrictions. About 59% of the survey participants carried out activities related to their studies in rooms that were used for purposes other than studying, such as living and leisure activities; 41.2% had their own separate study room; and 25.3% of the respondents had to coordinate the use of their learning place with other people living in the household. Almost three-fourths of the participants (74.7%) had access to their learning place at all times. Office desks were available to 69.6% of participants, while 38.5% studied on dining tables either exclusively or in addition to an office desk. Half had access to an office chair, while 43.2% used a living room chair as work seating either exclusively or additionally. Almost all participants reported having a laptop and/or a desktop computer as available equipment (99.2%). While 6.2% used a desktop computer only, laptops were the electronic devices most frequently used: 93% had a laptop available for studying, and 75.9% used a laptop without an additional desktop computer. About 69.3% used smartphones and 35.4% used tablets in addition to a laptop and/or desktop computer at their learning place. About 1% reported using only a tablet or a tablet in combination with a smartphone for online learning activities. Table 3 illustrates the proportionate availability of office equipment and IT infrastructure elements.

The analysis of the interviews yielded similar results. The living situations described in the interviews were all quite spacious, with a learning place either in a separate room or in a designated area. In most cases, the learning place was also used for the home office. In one case, the interviewee had a study room but preferred to conduct online learning activities at the kitchen table in a two-person household with an adult child. Two interviewees had to coordinate the use of their learning place with other household members, while the others did not. While three of the interview participants had ergonomic office furniture in their learning place, the others used ordinary kitchen tables and chairs and, in one case, antique furniture. Regarding technical equipment, all of the interviewees said that they used laptops primarily for distance learning, supplemented at most by a mouse and/or a headset and, in one case, by external speakers. Although several had additional IT equipment such as printers and scanners available, they stated that these were not used for their online learning activities.

### Perception of home learning environment for digitally-supported learning

In this section of the study, we examine how academic continuing education students perceived their home learning environment for digitally-supported learning during the initial COVID-19 restrictions. We investigated 11 attributes of spatial characteristics and indoor environmental conditions, with exploratory factor analysis (EFA) being conducted to discover possible dimensions (underlying factors). The extraction method of principal component analysis and varimax rotation with Kaiser normalisation was used to identify sub-scales that fit together statistically. The EFA produced a two-factor solution based on the varimax rotation with Kaiser normalisation and eigenvalues λ > 1. The two factors explained 55.2% of the variance. The Kaiser–Meyer–Olkin (KMO) measure of sampling adequacy was 0.853 and Bartlett’s Test of Sphericity was significant ($\chi^{2}$ (55) = 1073.03, *p* < 0.001). Table 4 shows the results of the factor analysis with factor loadings.

The two factors were interpreted as follows:

- Factor 1: Learning Place Quality (explaining 29.2% of variance)
- Factor 2: Indoor Environmental Quality (IEQ) (explaining 26.0% of variance).

Consistency of perceptual space was verified with Cronbach’s alpha. The values for the reliability coefficient for the two factors ranged from 0.75 to 0.83 (Table 5). Furthermore, for the items of the factors, the item selectivity (*r*_it_) and mean values (*M*) as well as standard deviations (*SD*) were calculated.

Students were asked about the extent to which their personal requirements were met regarding 11 different attributes. These describe the spatial characteristics and environmental conditions of their predominantly-used learning place and were allocated to one of two identified factors in the exploratory factor analysis (EFA), interpreted as F1 Learning Place Quality and F2 Indoor Environmental Quality. For most of the attributes, students rather agreed (3) or agreed (4) that their requirements were met (Table 6).

A rather high level of satisfaction (mean ± standard deviation) was found regarding perceived indoor air quality (good ventilation conditions, 3.71 ± 0.54), supply of daylight (3.66 ± 0.62), thermal comfort (comfortable temperature conditions, 3.63 ± 0.58) and technical equipment (3.51 ± 0.72). Only distraction-free environment (2.99 ± 1.02) and ergonomic aspects (ergonomic work-compatible furniture 2.68 ± 1.08) were reported by the students to be less than satisfactory, and there were greater variances in terms of satisfaction.

Interview participants were also satisfied with the spatial characteristics and equipment in the learning place. Sufficient space, quietness, light or brightness and proximity to nature were named as reasons for satisfaction with the living situation as well as with the physical learning environment. Except for the youngest interview partner, who mentioned that he wanted better IT equipment but could not afford it, the interviewees were quite satisfied with their IT equipment. Some expressed astonishment that participation in online learning activities worked well despite their rather basic technical equipment.

